# Role of α- and β-adrenergic signaling in phenotypic targeting: significance in benign and malignant urologic disease

**DOI:** 10.1186/s12964-021-00755-6

**Published:** 2021-07-20

**Authors:** M. Archer, N. Dogra, Z. Dovey, T. Ganta, H.-S. Jang, J. A. Khusid, A. Lantz, M. Mihalopoulos, J. A. Stockert, A. Zahalka, L. Björnebo, S. Gaglani, M. R. Noh, S. A. Kaplan, R. Mehrazin, K. K. Badani, P. Wiklund, K. Tsao, D. J. Lundon, N. Mohamed, F. Lucien, B. Padanilam, M. Gupta, A. K. Tewari, N. Kyprianou

**Affiliations:** 1 Department of Urology, Icahn School of Medicine at Mount Sinai, 6th Floor, 1425 Madison Avenue, New York, NY 10029 USA; 2 Department of Pathology and Molecular and Cell Based Medicine, Icahn School of Medicine at Mount Sinai, New York, NY USA; 3 Department of Genomic Sciences, Icahn School of Medicine at Mount Sinai, New York, NY USA; 4 Tisch Cancer Institute, Icahn School of Medicine at Mount Sinai, New York, NY USA; 5 Division of Hematology and Medical Oncology, Mount Sinai Hospital, New York, NY USA; 6 Department of Molecular Medicine and Surgery, Section of Urology, Karolinska Institute, Stockholm, Sweden; 7 Department of Medical Epidemiology and Biostatistics, Karolinska Institute, Stockholm, Sweden; 8 Department of Urology, Mayo Clinic, Rochester, MN USA; 9 Department of Oncological Sciences, Icahn School of Medicine at Mount Sinai, New York, NY USA

**Keywords:** Adrenoceptors, α- and β-adrenergic blockade, Urologic tumors, Cell polarity, Phenotypic landscape, Fibrosis, Kidney disease

## Abstract

The urinary tract is highly innervated by autonomic nerves which are essential in urinary tract development, the production of growth factors, and the control of homeostasis. These neural signals may become dysregulated in several genitourinary (GU) disease states, both benign and malignant. Accordingly, the autonomic nervous system is a therapeutic target for several genitourinary pathologies including cancer, voiding dysfunction, and obstructing nephrolithiasis. Adrenergic receptors (adrenoceptors) are G-Protein coupled-receptors that are distributed throughout the body. The major function of α1-adrenoceptors is signaling smooth muscle contractions through GPCR and intracellular calcium influx. Pharmacologic intervention of α-and β-adrenoceptors is routinely and successfully implemented in the treatment of benign urologic illnesses, through the use of α-adrenoceptor antagonists. Furthermore, cell-based evidence recently established the antitumor effect of α1-adrenoceptor antagonists in prostate, bladder and renal tumors by reducing neovascularity and impairing growth within the tumor microenvironment via regulation of the phenotypic epithelial-mesenchymal transition (EMT). There has been a significant focus on repurposing the routinely used, Food and Drug Administration-approved α1-adrenoceptor antagonists to inhibit GU tumor growth and angiogenesis in patients with advanced prostate, bladder, and renal cancer. In this review we discuss the current evidence on (a) the signaling events of the autonomic nervous system mediated by its cognate α- and β-adrenoceptors in regulating the phenotypic landscape (EMT) of genitourinary organs; and (b) the therapeutic significance of targeting this signaling pathway in benign and malignant urologic disease.

**Video abstract**

**Supplementary Information:**

The online version contains supplementary material available at 10.1186/s12964-021-00755-6.

## Introduction

The urinary tract is highly innervated by autonomic nerves. These nerves are essential in the development of the urinary tract, the production of trophic factors, and the regulation of homeostatic function [1–3]. The prostate gland is higly innervated due to its unique anatomical positioning that allows for facile manipulation of sympathetic nervous system (SNS) and parasympathetic nervous system (PSNS) signals [1]. Emerging evidence suggests that these neural signals may become dysregulated in several genitourinary (GU) organs and disease states, both benign and malignant [3, 4]. The current understanding of the mechanisms underlying α1-adrenoreceptor signaling, led to the identification of therapeutic benefits of blockade of these receptors in patients with urological diseases. a1-adrenoceptor antagonists are widely established as the first treatment choice to treat men with lower urinary tract symptoms (LUTS), most commonly associated with benign prostatic hyperplasia (BPH). The mechanism of action of α-blockade is a relaxation of smooth muscles in the bladder, urethra, and prostate by α1-adrenoceptor antagonists, which results in enhanced urinary flow and decreases LUTS.

Compelling evidence from diverse investigative teams, has implicated both α-and β- adrenergic signaling exerts regulatory control on critical cellular processes contributing to human cancer including, sustained proliferative signaling, resisting anoikis, inducing cell polarity and epithelial-mesenchymal transition (EMT), promoting angiogenesis, immune system evasion, promotion of local invasion and metastasis, cancer cell energy metabolism, increased inflammation and neovascularity of the tumor microenvironment (TME).

## Distribution of selective α- and β-adrenergic receptors (adrenoceptors)

Adrenergic receptors (adrenoceptors) are G-Protein coupled-receptors that are distributed throughout the body. They serve as receptors for catecholamines (noradrenaline and epinephrine) secreted from the autonomic sympathetic nervous system and adrenal medulla and play an important role in the regulation of a wide range of diverse physiological processes in human biological systems [5]. Alpha (α) adrenoceptors mediate smooth muscle contraction and vasoconstriction, while beta (β) receptors mediate vasodilation, smooth muscle relaxation, bronchodilation, and excitatory cardiac function [6, 7]. The α-adrenoceptors are divided into α1 and α2 subtypes. The α1 adrenoceptors play an important signaling role in all vasculature (including vasculature of the urinary tract) and mediate vasoconstriction (Fig. 2). Exogenous and endogenous epinephrine and norepinephrine activate these receptors through the Gq family of G proteins, which stimulates the hydrolysis of membrane phospholipids and subsequent generation of inositol phosphate and diacylglycerol (DAG) [8–10]. The binding of inositol phosphate to its receptor then leads to mobilization of calcium from intracellular storage sites and subsequent smooth muscle contraction [8]. Contrary to α1-adrenoceptoors, α2 adrenoceptors respond to epinephrine and norepinephrine through the Gi family of G proteins, which inhibits adenyl cyclase and subsequently results in inhibition of norepinephrine release from presynaptic neurons. Thus α2-adrenoceptors provide negative feedback on α1-adrenoceptors. While potentially important for their functional interactions with α1 adrenoceptors, α2-adrenoceptors have not been studied for in urologic disease [11].

The α1-adrenoceptors are further sub-divided into α1A, α1B, and α1D, with the α1A subtype of therapeutic interest in urologic pathophysiology because of its location in the prostate, vas deferens, and urethra in humans [7, 12–14] (Fig. 2). Additional evidence demonstrated the expression of α1A in the cortex, pelvis, calyces, blood vessels and tubules of the kidney [15–17]. The α1A-subtype predominantly regulates smooth muscle tone in the urethra, prostate and bladder neck [18, 19]. About 70% of the prostatic adrenoceptor mRNA expression has been found to be of the α1A-subtype, though all three subtypes are found throughout the prostatic epithelial and stromal components [20]. Notably, the majority of α1D-adrenoceptors were found in the detrusor muscle of the bladder, the bladder neck, and the sacral region of the spinal cord [19, 21, 22]. Prazosin was found to have lower affinity to lower urinary tract α1A-adrenoceptors than α1-adrenoceptors in other systems of the body, and thus this particular sub-classification of α1A-adrenoceptors in the lower urinary tract have been named α1L-arenorecptors. While pharmacologically distinct, α1L-adrenoceptors are products of the α1-adrenoceptor gene, but its altered phenotype is still under investigation [23].

The α1-adrenoceptor antagonists, doxazosin, tamsulosin, terazosin and prazosin, can alleviate urinary tract obstructions (LUTS) and treat voiding dysfunction by relaxing ureter, prostatic and bladder smooth muscle [24]. Moreover, given that these α1-adrenoceptors can induce gene expression of pro-inflammatory cytokines, such as interleukin 6 (IL-6) (driven by the α1A subtype), signal transducer and activator of transcription 3 (STAT3) and glycoprotein 130 (driven by α1A and α1D), one recognizes the magnitude of potential anti-inflammatory effects by α1-adrenoceptor blockade [25]. α1-adrenoceptor antagonists may also lead to inhibition of receptor activation on urothelium, vasodilation of blood vessels, and suppression of excitatory afferent nerve signaling throughout the urinary tract [26, 27]. Specifically, prazosin and tamsulosin effectively reduce the frequency of prostatic arterial contractions in pig models [27]. Studies using in vivo pre-clinical models of rats, demonstrated that the α1D-receptor antagonist naftopidil suppressed the excitatory effects induced by bladder distension and prolonged the intercontraction interval of bladder cells [26]. Interestingly α1-adrenoceptor blockade can induce synthesis of new α2-adrenoceptors, which subsequently override the post-junctional domain and function of α1-adrenoceptor antagonists. This effect has mostly been investigated in the context of treatment of hypertension and renal vascular denervation, as α2-adrenoceptors have been found to overcome sympathetic stimulation and increase sodium excretion from renal tubules [28]. In addition to α1-adrenoceptors, β-adrenoceptors mediate many of the effects of norepinephrine released from the sympathetic nervous system and epinephrine released from the adrenal medulla through the Gs family of G proteins that activate adenylyl cyclase [5, 6, 29]. While it is established that the β1 and β2 subtypes mediate most signaling in the urinary tract, emerging evidence defines the role of the third subtype β3 in inducing smooth muscle relaxation, with potential therapeutic targeting value for cancer treatment [29]. The β2 and β3 adrenoceptors are found throughout the GU tract in the ureter, bladder, prostate, and urethra, while β1 adrenoceptors are mainly present in the ureter and bladder [29].

In the human ureter, $\mathrm{\beta }$-adrenoceptors are involved in ureteral relaxation, though norepinephrine appears to mainly act on α1-adrenoceptors with subsequent contraction as opposed to relaxation in vitro and in vivo [30, 31]. However, the net catecholaminergic effect may depend on the specific segment of the ureter, as the distal segments exhibit greater β-adrenoceptor effects [32]. In the bladder, β-adrenergic stimulation can reduce symptoms of overactive bladder contractions. Bladder relaxation occurs in the detrusor of the bladder, as opposed to the bladder neck, and is driven predominantly by β3-adrenoceptors [29]. While β-adrenergic stimulation may have little effect on basal prostate tone, β2-adrenoceptors may inhibit α1-adrenoceptor-independent prostate contraction (Fig. 2) [33, 34]. In vitro studies have shown that norepinephrine stimulates cell growth, and progression of autochthonous prostate cancer mouse models, which in turn can be inhibited through β-adrenoceptor antagonism [4, 35, 36].

## Significance of targeting of adrenergic signaling mechanisms

α1-adrenoceptor antagonists are currently used for hypertension but have also been widely used to treat obstructing kidney stones by decreasing intra-ureteral pressure and increasing fluid passage [37]. The clinical efficacy of α1-adrenoreceptor antagonists in the treatment of benign prostatic hyperplasia (BPH) and prostate cancer has been attributed to the ability of these agents to not only reduce smooth tone and relieve overall obstruction, but to also induce apoptosis and overcome anoikis resistance [38–42]. The quinazoline-derived α1-antagonists induce apoptosis and overcome anoikis resistance in human castration-resistant prostate cancer (CRPC) cells and androgen-sensitive prostate cancer cells, suggesting a strong molecular rationale for the use of these compounds as anti-tumor agents [43–45]. Indeed rapidly expanding evidence supports the therapeutic value of these FDA-approved drugs for the treatment of prostate, bladder, colorectal, and renal cell carcinoma [38, 40, 43, 46, 47]. As schematically illustrated on Fig. 3, the mechanisms of apoptosis induction involve: 1) Smad activation of transforming growth factor (TGF)-β1 signaling, which controls cell proliferation, apoptosis and EMT in several cell types [47, 48], 2) transcription of the NF-κB inhibitor IκBα, 3) activation of the death receptor Fas-assosciated death domain (FADD)-mediated caspase-8 activation [47, 48]. The α1-adrenoceptor antagonists can impair VEGF-induced angiogenesis and target Akt survival mechanisms [47, 49]. At the cellular level, α1-adrenoceptor antagonists have the ability to:(a) block cellular adhesion and invasion by targeting cell–cell interaction and impairing cell tight junctions (and also between epithelial and endothelial cells with the extracellular matrix [ECM]); (b) functionally drive the phenotypic interconversions between epithelial-mesenchymal-transition (EMT) to mesenchymal-epithelial-transition (MET); and (c) increase cellular vulnerability to death via anoikis [49] (Fig. 3). Additional mechanistic targets have been identified involving integrin-linked kinase (ILK), a serine and threonine protein kinase, that plays a key role in anoikis resistance by interacting with the cytoplasmic domains of β1-integrin and β3-integrin, which are pivotal in regulating cell adhesion, fibronectin-ECM assembly, and anchorage-dependent cell growth [50–54]. Within the tumor microenvironment, ILK is activated in its phosphorylated form by focal adhesion kinase (FAK) and phosphatidylinositol 3-kinase (PI3-kinase)/Akt pathways [50, 55, 56]. By inhibiting ILK, quinazoline-derived α1-adrenoceptor antagonists disrupt these cell-survival signals towards anoikis induction [57, 58]. As resistance to anoikis is one of the major hallmarks of metastatic cancer cells, the ability to prevent such resistance is a distinct therapeutic benefit afforded by α1 adrenoceptor antagonists.

Due to the effects in relaxing smooth muscles in the ureter and prostate, β2 and β3 adrenoceptor agonists have been implicated in targeting ureteral stones [29, 59]. Compelling mechanistic evidence suggests that β adrenoceptor antagonists exert anti-neoplastic effects by suppressing the MAPK pathway, modulating inflammation and oxidative stress, and inhibiting of nitric oxide synthase (NOS) expression.[6] The use of video-microscopy and microarray analysis demonstrated that neurotransmitters like norepinephrine can induce tumor cell migration in human prostate cancer cells. Because norepinephrine acts mostly via β2 receptors in the prostate gland, β-adrenoceptor antagonists may serve as a potential preventative measure for metastasis [60]. Clinically, the long-term usage [3–5] of atenolol may be beneficial in reducing the risk for prostate cancer, as compared to those not on β-adrenoceptor antagonists or those taking other β-adrenoceptor antagonists, such as carvedilol and metoprolol [61]. A systematic literature review and meta-analysis of 16,825 patients found that this β-blocker use was associated with lower cancer-specific mortality in patients with prostate cancer. Furthermore, β-adrenoceptor antagonists were reported to increase anti-angiogenic effects of chemotherapeutic regimens as well as prevent metastases in breast and ovarian cancers [62].

**Fig. 1:**
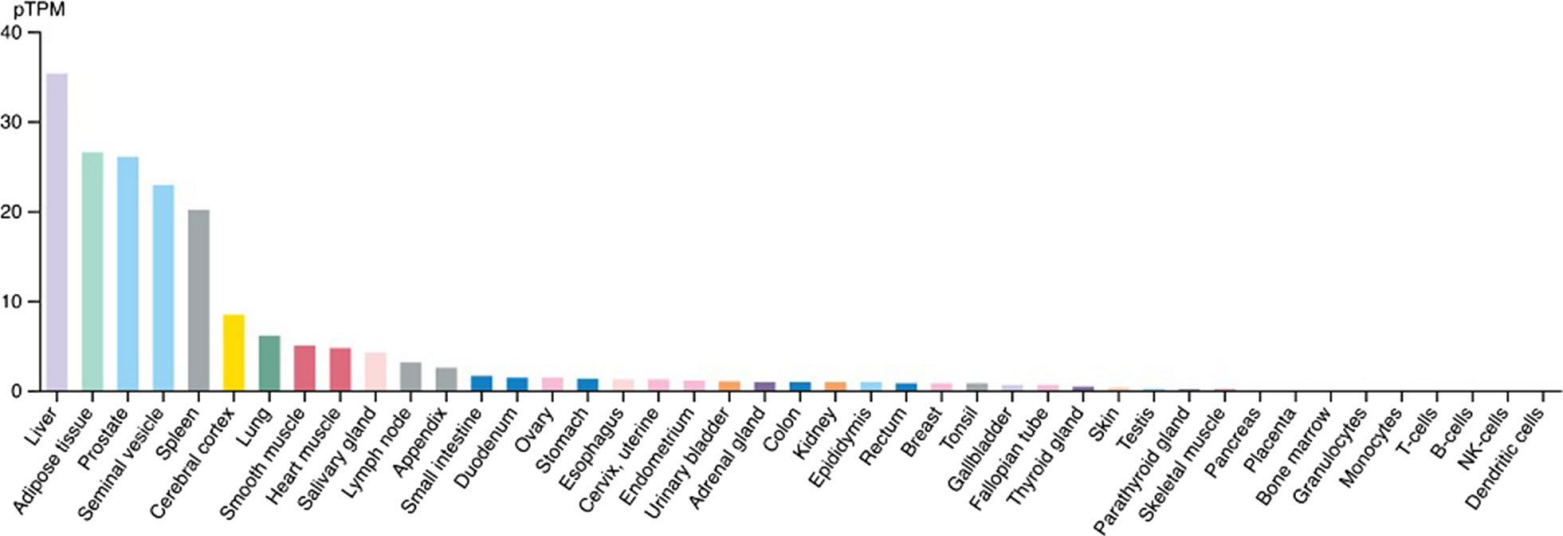
RNA expression profile of α1- adrenoceptors from a data set in 43 human tissue types. In GU organs, high expression of α1A-adrenoceptors is detected in the prostate and seminal vesicles, while urinary bladder and kidney exhibit significantly lower levels of mRNA for α1A- adrenoceptor expression

Adrenoceptors serve as receptors for catecholamines (noradrenaline and epinephrine) secreted from the autonomic sympathetic nervous system and play an important role in the regulation of a wide range of physiological systems in the body (Fig. 1) [5]. Though both autonomically innervated, α- and β-adrenoceptors largely mediate alternative functions. α- adrenoceptors mediate smooth muscle contraction and vasoconstriction, while β-adrenoceptors mediate vasodilation, smooth muscle relaxation, bronchodilation, and excitatory cardiac function [6, 7].

Table 1 summarizes the ubiquitous nature of α- and β‐adrenoceptor mRNA expression in the human body and their distribution in specific tissue types [63, 64]. The consequential receptor-mediated signaling in a tissue-specific pattern regulating diverse physiological processes, presents a rational targeting platform by pharmacological intervention, thus the clinically available, the Food and Drug Administration (FDA) approved α-and β-adrenoceptor antagonists [63]. In this context, comparative RNA expression analysis of adrenergic receptors in 37 tissue types from human protein atlas (HPA) tissue gene expression profiles to put forth a combined mapping of the adrenoceptors in the human body [65], revealed that human liver, adipose tissue, prostate, seminal vesicles contain the most abundant α1A adrenoceptor RNA expression (Table 1) [65]. The adrenoceptor α1D, however, was highly enriched in cervix, uterine, and prostate. The β-adrenoceptors were primarily abundant in placenta, ovary, heart, prostate, and bladder (Table 1). This in-depth comparison provide a rational for why α-adrenoceptor antagonists have been beneficial for the treatment of BPH, erectile dysfunction, and heart failure [63], while β-adrenoceptor antagonists are specifically beneficial to cardiac arrhythmia and Angina pectoris [66]. Consequently, BPH, prostatitis, and obstructing ureteral stone are effectively treated with α1-adrenoceptor antagonists [67]. Significantly, β-adrenoceptor antagonists are used for the treatment of patients with aortic aneurysm, cardiovascular abnormalities, and BPH.

**Table 1:** α- and β-adrenoceptor distribution and expression in different human tissues

Subtypes of receptors functional in adrenergic signaling in target tissues	Adrenoceptor subtype	Tissue RNA expression and distribution in different organs
α-adrenoceptors	α-1A adrenoceptor	1. Liver 2. Adipose tissue 3. Prostate 4. Seminal vesicles 5. Spleen
	α-1B adrenoceptor	1. Spleen 2. Cerebral cortex 3. Liver 4. Ovary 5. Kidney
	α-1D adrenoceptor	1. Prostate 2. Cervix, uterine 3. Seminal vesicles 4. Spleen 5. Fallopian tube
β-adrenoceptors	β-B1 adrenoceptors	1. Placenta 2. Heart 3. Lung 4. Prostate 5. Cerebral cortex
	β-B2 adrenoceptors	1. T-cells 2. Granulocytes 3. Monocytes 4. NK-cells 5. Lung
	β-B3 adrenoceptors	1. Ovary 2. Placenta 3. Gallbladder 4. Urinary bladder 5. Epididymis

Comparative cell type localization and presence of different subtypes of **α-and β-**adrenoceptors in different tissue types of human body. Top 5 ranked tissue by RNA expression are shown

Thus one may argue that the relative distribution of adrenergic receptors is unique to tissue types (Fig. 1) and in-depth analyses of the target receptors followed by their subsequent mapping may provide a rationale of targeting both α- versus β- adrenoceptors for urological diseases. This goal is benefited with the advent of novel genomic and proteomic technologies being applied in prostate and renal cancer [68]. We are entering in to a new area of large scale data analyses, which may help find better adrenergic targets for the next-generation therapeutic interventions of urological diseases [68, 69].

α1-adrenoceptor antagonists are currently used for hypertension but have also been widely used to treat kidney stones by decreasing intra-ureteral pressure and increasing fluid passage [37], BPH and more recently to suppress prostate tumor growth and vascularity [38–42]. Quinazoline-derived compounds are not only effective in inducing apoptosis in benign prostate epithelial cells but also in both castration-resistant prostate cancer (CRPC) cells and androgen-sensitive prostate cancer cells [43–45] Moreover these α1-antagonists have been found effective in the treatment of other GU tumors including, bladder cancer, colorectal cancer, and renal cell carcinoma.[38, 40, 43, 46, 47].

Growing evidence suggests the pro-apoptotic effects of quinazoline-derived α-adrenoceptor antagonists against prostate cancer epithelial and endothelial cells, mechanistically via inducing apoptosis via activation of transforming growth factor (TGF)-β1 signaling, which controls EMT and differentiation, in diverse cell types,[47, 48], transcription of the NF-κB inhibitor IκBα, and engaging the death receptor Fas-associated death domain (FADD)-mediated caspase-8 activation,[47, 48]. The action of these antagonists against tissue vascularity proceeds via inhibition of the vascular endothelial growth factor (VEGF)-induced angiogenesis and Akt survival mechanisms [47, 49]. Furthermore, α1-adrenoceptor antagonists have the ability to block adhesion, migration, and invasion due to loss of appropriate interaction between epithelial and endothelial cells with the ECM, thus increasing anoikis vulnerability [49]. Considering that resistance to anoikis is hallmark feature of the metastatic journey of cancer cells, the ability to overcome such resistance provides a distinct therapeutic benefit by α1-adrenoceptor antagonists towards impairing metastasis.

Due to the effects in relaxing smooth muscles in the ureter and prostate, the β2 and β3 adrenoceptor agonists have been found to be therapeutic in the treatment of ureteral stones; β3 adrenoceptor agonists can specifically inhibit prostatic smooth muscle contraction and reduce secondary to BPH symptoms [29, 59]. Pharmacologic targeting of β-adrenoceptor signaling results in tumor suppression by inhibiting the MAPK signaling pathway, modulating inflammation and oxidative stress [6] (Fig. 3). The use of video-microscopy and microarray analysis demonstrated that neurotransmitters like norepinephrine induces tumor cell migration in prostate cancer cells, supporting the use of β-blockers as a preventative measure for prostate cancer metastasis [60]. Clinically, the long-term usage [3–5] of atenolol may be beneficial in reducing the risk for prostate cancer, compared to those not on β-adrenoceptor antagonists or those taking other β-adrenoceptor antagonists, such as carvedilol and metoprolol [61]. A systematic literature review and meta-analysis of 16,825 patients found that exposure to β-adrenoceptor antagonists was associated with lower cancer-specific mortality in patients with prostate cancer. Significantly, β-adrenoceptor antagonists can enhance the anti-angiogenic effects of chemotherapeutic regimens and prevent metastases in breast and ovarian cancers [62].

## Pharmacologic targeting of adrenergic signaling in benign urologic disease

**Fig. 2:**
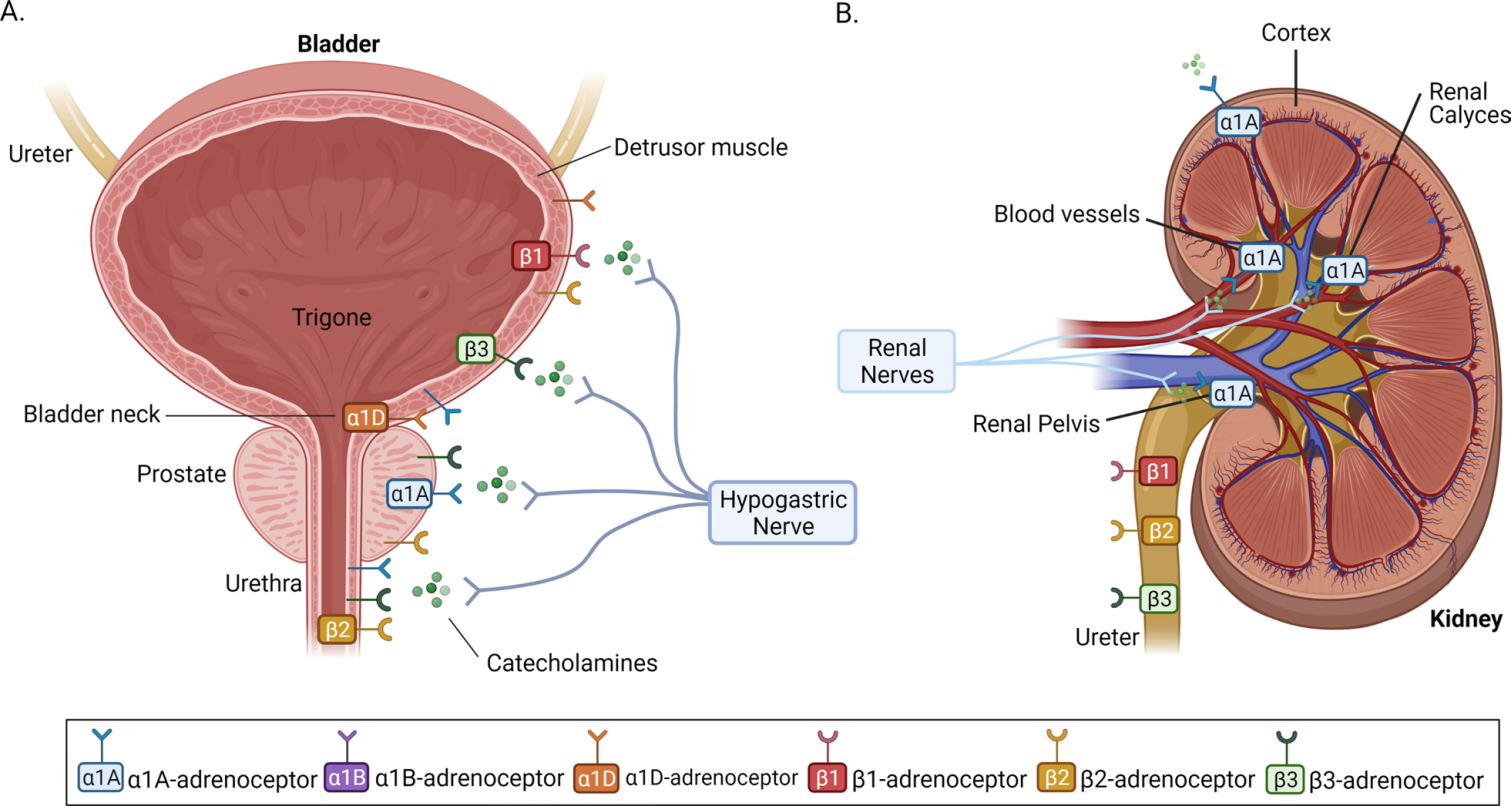
α- and β- adrenoceptor Expression and Distribution in the Urinary Tract. Schematic representation of the location and distribution of α and β -adrenoceptors and their subtypes in the bladder, prostate gland and urethra (**a**), and the kidney and ureter (**b**). The α-adrenoceptors are divided into α1 and α2 subtypes. The α1 receptors are important receptors in the urinary tract vasculature via their ability to mediate vasoconstriction. Exogenous and endogenous epinephrine and norepinephrine activate these receptors through the Gq family of G proteins, which stimulates the hydrolysis of membrane phospholipids and subsequent generation of inositol phosphate and diacylglycerol (DAG). α and β-adrenoceptors respond to signals from catecholamines (noradrenaline and epinephrine) secreted from the autonomic sympathetic nervous system through the hypogastric and renal nerves to regulate physiological functions and cellular processes. (Insert, color-coding key illustrating the various adrenoceptor subtypes)

Pharmacologic targeting of α- and β-adrenoceptors is clinically applied in the treatment of benign urologic illnesses. Most commonly, α-adrenoceptor antagonists are used in the treatment of benign prostatic hyperplasia (BPH). As men age, the prostate undergoes benign growth which may obstruct the flow of urine or alter bladder dynamics leading to lower urinary tract symptoms (LUTS) [70, 71]. α1A adrenoceptors are the most common subtype within the prostate functioning to facilitate smooth muscle tone[72] (Fig. 2). The α1-adrenoceptor antagonists target this receptor signaling, resulting in prostatic smooth muscle relaxation, which alleviate prostatic obstruction and improve urinary flow [73]. Tamsulosin, a selective α1A adrenoceptor antagonist [74], is employed as a first line pharmacotherapy for LUTS in men and is one of the most commonly prescribed medications in the US [75].

Αlpha (α)-adrenoceptors are also found in the bladder and similarly facilitate smooth muscle tone [73, 76]. Regarding voiding function, relaxation of the bladder’s detrusor muscle has two opposing functions depending on anatomic location. The most common α1adrenoceptor subtype in the bladder is the α1d adrenoceptor [76], making for a therapeutic target in patients with storage-type LUTS. Indeed, naftopidil, a selective α1d receptor antagonist, has shown efficacy in improving urinary storage symptoms [77]. The use of α-blockers for LUTS symptoms has been associated with ejaculatory dysfunction believed to be secondary to a reduction in seminal fluid emission due to central nervous system sympathetic inhibition [78].

In addition to the treatment of voiding dysfunction, α1-adrenoceptor antagonists are routinely used in the management of kidney stone disease. As discussed earlier α-adrenoceptors are expressed throughout the ureter and the lower urinary tract, functionally modulating smooth muscle tone [79, 80]. The use of α-adrenoceptor antagonists to relax smooth muscle within the ureter in patients with obstructing ureteral stones may increase luminal diameter and facilitate spontaneous stone passage (Fig. 2). The distal ureter has a greater muscular component and greater α-receptor expression than the proximal ureter [79, 80]. Accordingly, α1-blockade is most clinically beneficial for medical expulsive therapy in patients with obstructing distal ureteral stones [81]. Similarly important is the use of α-adrenoceptor antagonists for the treatment of ureteral stent colic. Ureteral stents are regularly used to alleviate upper urinary tract obstruction; this approach however is often associated with severe discomfort causing a condition known as “stent colic.” Αlpha-adrenoceptor antagonists have been shown to alleviate symptoms of stent colic, likely secondary to their regulatory (relaxation) effect on the ureteral smooth muscle [82]. The most common receptor subtype within the human ureter is the α1a adrenoceptor, making tamsulosin the α1-antagonist of choice for the treatment of stent colic [80].

Beta-3 sympathetic receptors are expressed in the detrusor muscle of the human bladder and are a pharmacologic target for the treatment of overactive bladder (OAB) [83]. Historically, the mainstay of pharmacotherapy for OAB was anticholinergic medications which indirectly increased relative sympathetic tone by blunting parasympathetic nervous system input via antagonism of muscarinic receptors [84]. However, anticholinergic medications have several side effects such as dry mouth, dry eyes, and constipation and have poor long-term adherence for many patients [84, 85]. Mirabegron, a medication approved a decade ago by the FDA, functions as a β3 selective adrenoceptor agonist that promotes detrusor relaxation and in turn alleviates symptoms of OAB [86]. β3 adrenoceptors are a common subtype within the human bladder [87] and the selective nature of mirabegron may explain the greater tolerability of mirabegron when compared to anticholinergics in patients with OAB [88]. As our understanding of sympathetic receptor expression within the urinary tract continues to evolve, our therapeutic armamentarium for the treatment of benign urologic disease will likely include β-blockade.

In the kidney, the expression and distribution of α1-adrenoreceptors are in the cortex, pelvis, calyces, blood vessels and renal tubules, suggesting potential effects of α1 adrenoceptor antagonists in renal pathophysiology [89–91]. The α1- and α2-adrenoceptors are present in the renal vasculature and mediate vasoconstriction of exogenous and endogenous noradrenaline [92].

Adrenoceptors are involved in both acute kidney injury (AKI) and chronic kidney disease (CKD) pathogenesis. Modulation of either α- or β-adrenoceptors protects renal ischemia/reperfusion injury (IRI) and nephrotoxic AKIs [93, 94], suggesting fine-tuning of its activity as a promising target to develop effective treatment for these clinical entities. α_2_-adrenoceptor blockers, yohimbine and atipamezole, and JP-1302, an α_2C_-adrenoceptor specific inhibitor, attenuate renal tubular damage in rat models of renal mass reduction and in mice subjected to unilateral IRI [95, 96]. In sharp contrast, activation of α_2A_-adrenoceptor using α_2A_-adrenoceptor agonists, clonidine and moxonidine inhibit activation of sympathetic nerves, resulting in protection against IRI, in unilateral IRI rabbits and rats that removed the contralateral kidney [97, 98], suggesting injury-dependent mechanism of α_2_-adrenoceptor antagonism. In the cisplatin AKI, yohimbine and JP-1302, α_2_-adrenoceptor antagonists, or nebivolol, β1-adrenoceptor antagonist, suppress renal tubular damage and dysfunction through reduction of plasma norepinephrine (NE) levels and inhibition of pro-inflammatory cytokine expression [99, 100].

A number of clinical and experimental studies suggest that maladaptive recovery from AKI predisposes to CKD progression [101–103]. A recent meta-analysis revealed that hospitalized patients experiencing AKI have a higher risk for CKD and cardiovascular complication [101]. Experimental studies from our group and other investigators, demonstrated that blockade of either β-adrenoceptor by propranolol, or α_2_-adrenoceptor by atipamezole, or α_2C_-adrenoceptor subtype by JP-1302 prevents long-term sequelae of ischemic AKI, including chronic inflammation and fibrosis [95, 104, 105]. Pharmacological inhibition of α_2_-adrenoceptor subtype(s) markedly suppressed unilateral ureteral obstruction (UUO)- or IRI-induced kidney fibrosis, along with reduced tubular injury and inflammation [95, 106]. In a CKD model of subtotal nephrectomy, inhibition of either α_1_-adrenoceptor or β-adrenoceptor mitigates the renal injury, and a combinational inhibition of α_1_- and β-adrenoceptors shows additive beneficial effects in renal injury [107].

Blockade using doxazosin, an α_1_-adrenoceptor antagonist, and atenolol, a β_1_-adrenoceptor antagonist blunts obesity-associated hypertension and increase of heart rate in both clinical and experimental studies [89–91, 108]. Diabetic kidney disease is the main cause of CKD and end-stage kidney disease, commonly accompanied by hypertension and additional cardiovascular complications [109, 110]. Beta (β)-blockers attenuate albuminuria, hypertension, and cardiovascular complications in patients with diabetic nephropathy [92]. β1‐cardioselective adrenoceptor antagonists, metoprolol and atenolol, recommended as antihypertensive agents in CKD or ESKD patients with hypertension, effectively lower blood pressure (BP) without significant decline of glomerular filtration rate (GFR) and renal blood flow (RBF) [92, 111–113]. Peripherally acting α-blockade against vascularity of vessel walls can be used either as a part of combination therapy or first-line therapy for the management of CKD and hypertension [114–116].

In kidney diseases, the beneficial effect of adrenoceptor antagonists is most likely associated with suppression of renal inflammation, tubular cell cycle arrest, cell death, and fibrosis. Upon kidney injury, inflammatory cells including monocytes/macrophages are recruited into the site of injury and secrete proinflammatory/fibrogenic cytokines, resulting in persistent tubular injury and fibrosis [117]. Monocytes, macrophages as well as renal tubular cells expresses most adrenoceptor subtypes, including α_2_-adrenoceptor. Activation of α_2_-adenoceptor in macrophage upregulates pro-inflammatory cytokines like tumor necrosis factor-α (TNF-α) and interleukin-6 (IL-6), while that of β_2_-adrenoceptor confers anti-inflammatory response [118]. Inhibition of α_2_-adrenoceptors prevented interstitial fibrosis in IRI and UUO models, along with reduction of profibrotic factors, TGF-β1 and Smad3, and thus suppressing myofibroblast activation and collagen deposition [95, 106]. In line with our results, α_2_-AR agonist upregulates TNF-α in macrophages [119], accelerates mesenchymal cell apoptosis [120] and triggers cell cycle arrest in oligodendrocyte progenitors [121]. Contrary to the results from kidney, blockade of α_2A_-adrenoceptor and α_2C_-adrenoceptor subtypes increases the susceptibility to develop heart failure after chronic pressure overload in mice [122, 123], suggesting tissue-dependent role of α_2_-adrenoceptors.

Another potential mechanism for α-adrenoceptor antagonism in the AKI and CKD model is associated with nitric oxide (NO) availability [124]. In the kidney, NO has numerous physiological roles including the modulation of renal sympathetic nerve activity [125, 126]. Renal NO synthase activity is reduced in both AKI and CKD [127–129]. Downregulation of NO synthesis are prevented by prior administration of an α_2_-adrenoceptor antagonist [130, 131] Administration of an α_2_-adrenoceptor agonist, B-TH933 to the denervated kidney restores both glomerular and tubular injury due to inhibition of NO [132]. Decline of NOS activity by L-NAME, a non-selective inhibitor of NOS, was significantly restored by quinazoline-based α_1_-adrenoceptors antagonists, prazosin, or doxazosin, and mitigated the renal injury [133–135]. Furthermore, it was also shown that β-adrenoceptor antagonists induce relaxation of renal vascular and glomerular endothelial cells [136, 137], while selective β-adrenoceptor blockers, such as carvedilol and nebivolol, protect gentamycin-induced nephrotoxicity, nephrectomy-induced CKD, or hypertensive CKD through upregulation of NO release [138–141].

The renin-angiotensin system (RAS) interacts with renal sympathetic nerve-derived signaling [142]. Renin that catalyzes cleavage of angiotensin (Ang) to Ang II release from the juxtaglomerular apparatus of the kidney and acts as a determinant of renal damage [89, 91]. Conversely, Ang II can enhance NE level by acting on sympathetic nerve terminals [143, 144]. Moreover, genetic inhibition of α_2A_-adrenoceptor or pharmacological inhibition of α_2_-adrenoceptor attenuates Ang II-mediated norepinephrine release in isolated kidneys with nephrectomy-induced CKD [145]. However, exploitation of the α-adrenoceptor signaling in the regulation of intrarenal RAS directly in benign kidney disease is ongoing.

## Significance of adrenergic signaling in phenotypic landscaping of the microenvironment

**Fig. 3:**
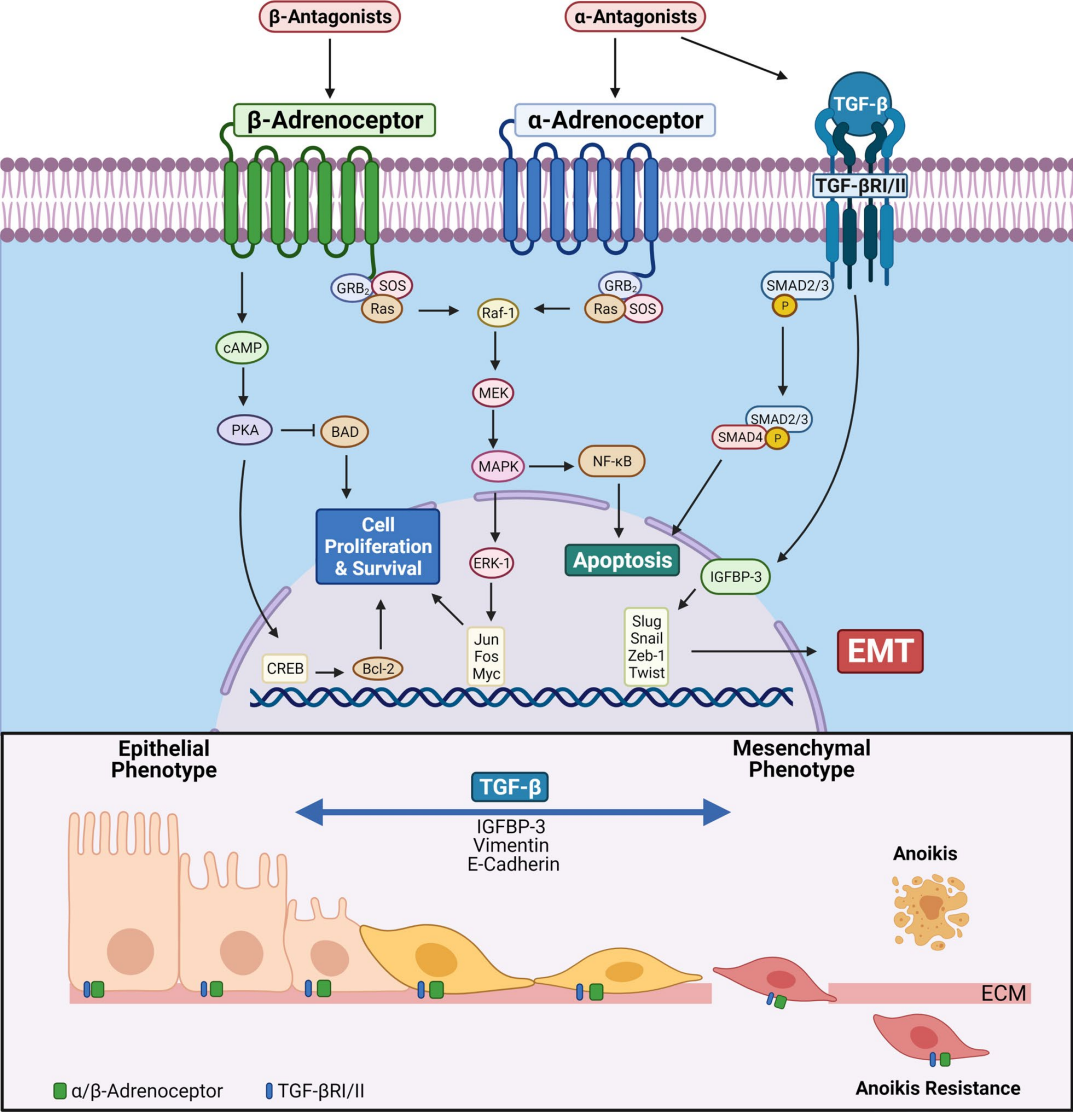
Molecular regulation of the phenotypic landscape by targeting α- and β-adrenoceptor signaling pathways. Top panel, Mechanistic signaling of α- adrenoceptor signaling and β-adrenoceptor eliciting effects on phenotypic EMT, apoptosis and cell proliferation. The α1-adrenoceptor signaling, activates the survival pathways NFκB and MAPK inducing transcriptional activation of growth genes (fos, myc). In response to α1-adrenoceptor pharmacologic targeting, in addition to blocking the α-adrenoceptor pathway, there is also inhibition of the TGF-β/ TGF-β receptor RI/RII/Smad signaling that induces apoptosis as well as phenotypic EMT. In response to β-adrenoceptor signaling there is activation of protein kinase A mechanism (though cAMP), promoting cell proliferation and survival via CREB activation and Bcl-2 overexpression, respectively. Lower panel, Impact of pharmacologic targeting of α1-adrenoeptor antagonists of the phenotypic interconversions (mesenchymal-EMT to epithelial-MET) within the tumor microenvironment landscape via regulation E-cadherin, IGFBP-3 and vimentin. Upon detachment from the ECM, cells unable to enter the phenotypic interconversion cycle mediated by TGF-β undergo anoikis. This provides the molecular targeting platform for α-adrenoceptor antagonists in tumor cells in the various GU organs, including the prostate and kidney

Pharmacologic targeting of α1-adrenoceptors in GU malignancies may proceed through induction of apoptosis. Quinazoline-based α1-adrenoceptor antagonists have been found to suppress growth of androgen-dependent and castration resistant prostate cancer [146] as well as renal cancer cells through induction of the extrinsic mechanism of apoptosis [45, 147]. As illustrated on Fig. 3, α-adrenoceptor antagonists can dictate the canonical TGF-β receptor signaling mechanism to induce apoptosis and EMT [43–45, 148]. Other α-adrenoceptor (quinazoline-based) antagonists including prazosin promote apoptotic activity by inducing cell cycle arrest as a result of DNA strand breaks, phosphorylation of cyclin-dependent kinase (CDK) causing CDK1 inactivation and G2 checkpoint arrest [149]. Novel quinazoline-based α-antagonists, (DZ-50) have been developed by our group, impair primary growth and progression of renal and prostate cancer to metastasis by inhibiting focal adhesion kinase (FAK) phosphorylation and protein kinase B (AKT) signaling pathways by inducing anoikis. This is consequential to the disruution of adhesion to the ECM via targeting the integrin β1 focal adhesion complexes (Fig. 3) [47, 150]. Further mechanistic evidence suggests that piperazine based α1-adrenoceptor antagonists may have an anti-tumor effect on cancer cells in vitro and in vivo*.* Naftopidil induced apoptosis in bladder cancer cell lines and xenograft tumors by activation of the caspase-3 cascade [151]. In contrast, sulfonamide-based α-adrenoceptor antagonists (tamsulosin) failed to induce apoptosis in prostate cancer cells suggesting that the chemical structure of α-adrenoceptor antagonists is functionally an important factor driving the apoptotic action of the drugs [45].

In human androgen-sensitive prostate cancer cells, that continuous exposure to catecholamine epinephrine is protective against apoptosis [152]. Through binding of agonists to β-adrenoceptor, there is upregulation of intracellular cyclic adenosine monophosphate (cAMP) to bind to PKA, leading to the phosphorylation of the cAMP responsive element binding protein (CREB) [153]. Stimulation of CREB activity induces expression of survival protein B-cell lymphoma 2 (Bcl-2) [153, 154]. The cAMP/PKA signaling further increases resistance to apoptosis by inhibition of the Bcl-2-associated death promotor (BAD) in in vitro and in vivo models of prostate cancer [153–155]. While the apoptotic effect of β-adrenoceptor antagonists has not been investigated in urologic tumors, there is evidence suggesting the ability of β-adrenoceptor antagonists, such as propranolol, to induce apoptosis in other cancers. Studies utilizing liver cancer and melanoma cell lines demonstrated that treatment with propranolol induced cell cycle arrest and apoptosis [156, 157]. In late stage breast cancer patients, neoadjuvant treatment with propranolol decreased the expression of survival protein Bcl-2 and increased tumor suppressor P53 in tumor specimens [158].

The adrenergic connection to angiogenesis and tumor vascularity has been defined at both the α- and β-adrenoceptor level. Angiogenesis under normal physiological conditions is primarily involved in the ovarian cycle and in wound healing and repair, existing only transiently in adults as their complete and mature vasculature is derived from the network of blood vessels formed during embryonic development [153, 159]. Angiogenesis supports tumor growth by establishing a network of blood vessels that provide nutrients and oxygen as well as waste removal to cells within the tumor microenvironment [160]. Adrenergic nerves are situated closely to the arterioles and capillaries within the stromal component of tissues, thus are ideally positioned to contribute to vasculature stimulation [1]. Angiogenesis occurs when the balance between pro- and anti-angiogenic factors is tipped in favor of supporting growth, an event known as the “angiogenic switch” [4, 161]. A hypoxic environment is created that stimulates transcriptional activity of hypoxia-inducible factor 1 (HIF1), driving transcription of pro-angiogenic genes [160]. Prominent pro-angiogenic factors include members of the VEGF family, transforming growth factors (TGF)-α and TGF-β, TNF-α, platelet-derived endothelial growth factor and fibroblast growth factor (FGF) [161, 162]. Inhibitors of angiogenesis include angiotensin, plasminogen activator-inhibitor-1, and several cytokines and metalloproteases [161]. β-Adrenoceptor signaling contributes significantly to enhanced tissue vascularity via activating the above pathways and promoters of anhiogenesis in human solid cancers, including GU tumors [162].

The human prostate gland is highly innervated [154], and is ideal as a model to study the coontribution of innervation to cancer development, as it is positioned fully enabling for facile manipulation of SNS and PSNS signaling [1]. Furthering this notion is the fact that the majority of prostate tumors emerge from the peripheral zone, a region that contains the highest abundance of nerves for the organ [154]. Within the TME, β-adrenoceptors are stimulated via adrenergic signals delivered by sympathetic nerves (Fig. 1.) [4]. Seminal work by Zahalka et al. First established that the expression of β2-adrenoceptors within prostate epithelial cells is necessary and critical to maintaining them at the proper metabolic state (i.e. performing aerobic glycolysis) essential for angiogenesis [4]. Orthotopic implantation of human prostate cancer xenografts (derived from PC-3 cells) revealed that xenografts grown in mice expressing WT β2-adrenoceptor grew exponentially post-day 18 compared to xenografts implanted in β2-adrenoceptor and β3-adrenoceptor-knockout (KO) mice [4]. Furthermore when sympathectomy or conditional cell-specific deletion of β2-adrenoceptor in endothelial cells was performed, angiogenesis was inhibited via upregulation of cytochrome C oxidase assembly factor 6 (Coa6), a protein involved in the electron transport chain and oxidative phosphorylation [1, 4]. Recent insights into the functional role the β2-adrenoceptor plays in angiogenesis in prostate cancer revealed a dose-dependent increase in VEGF expression in LNCaP human prostate cancer cells, upon stimulation of β2-adrenoceptor via epinephrine [154]. In an independent study, Park et al. observed similar upregulation in VEGF expression levels, as well as expression of hypoxia-inducible factor (HIF)-1α in androgen-independent human prostate cancer cells PC-3, treated with norepinephrine and isoproterenol, an effect that was mechanistically mediated through increased activity of the cAMP/PKA pathway [154, 163]. Conditioned media from prostate cancer cells exposed to norepinephrine lead to capillary tube formation [164], while in vivo treatment of rats with propranolol significantly reduced the ventral prostate volume [154, 164].

## Adrenergic signaling controls epithelial mesenchymal transition (EMT) and cell polarity

A rapidly expanding body of work has shed the light on the molecular and cellular mechanisms involved in the cross-talk between α- and β-adrenergic signaling and EMT [41, 165]. Phenotypic EMT is a tightly regulated cellular process directed by TGF-β signaling and where cancer cells adopt migratory and invasive properties including loss of intercellular junctions, anchorage-independent survival, and metalloprotease-dependent matrix degradation [166]. EMT has been recognized as an important functional contributor the increased number of myofibroblasts in cancer and fibrotic diseases. Renal sympathetic nerve activity is elevated in patients and pre-clinical CKD models and contributes to renal interstitial fibrosis in obstructive nephropathy. The underlying mechanisms of sympathetic overactivation in renal fibrosis remain revealed that norepinephrine (NE), the main sympathetic neurotransmitter, promotes TGF-β1-induced EMT. Of direct translational significance is recent evidence on the impact of pharmacological targeting of α1-adrenoceptors on reducing the kidney injury and renal fibrosis in a unilateral ureteral obstruction pre-clinical model by suppressing EMT in the kidneys [167]. Thus, sympathetic overactivation facilitates EMT of renal epithelial cells and fibrosis via the α1-adrenoceptor driven TGF-β signaling mechanism, inhibition of which by an α1-adrenoceptor antagonist provides a promising approach for the treatment of renal fibrosis (Fig. 3).

EMT has been shown to play a role in airway remodeling induced by eosinophils. Procaterol, a selective and β_2_-adrenoceptor agonist, clinically for asthmatic attacks as a controller, was recently shown to functionally suppress EMT of airway epithelial cells induced by eosinophils [168]. Mechanistic studies revealed that Butoxamine, a specific β_2_-adrenoceptor antagonist, significantly abrogated changes induced by procaterol. In addition, procaterol inhibited expression of adhesion molecules induced during the interaction between eosinophils and bronchial epithelial cells, supporting the involvement of adhesion molecules in EMT. Procaterol significantly inhibited in vitro associated morphological changes of bronchial epithelial cells, decreased vimentin, and increased E-cadherin expression [168].

Released by sympathetic nerves or through leakage from vascular neuromuscular junctions, catecholamines are found in high concentrations within the TME. Through binding of β-adrenoceptors on tumor cells, catecholamines have been shown to promote integrin-dependent cell adhesion, calcium-dependent cytoskeleton remodeling, formation of invasive protrusions (invadopodia) and release of ECM-degrading proteases [169–176]. While the functional role of β-adrenoceptor signaling has been investigated in cancer, less is known about the pro-tumorigenic role of α-adrenoceptors. Recently, preclinical and clinical data have supported the antitumor action of α-adrenoceptor antagonists on bladder cancer growth and migration and extended progression-free survival in bladder cancer patients [177]. Pharmacologic blockade of α-adrenoceptor signaling is capable of reversing EMT to MET by targeting insulin-like growth factor binding protein-3 (IGFBP3) and the TGF-β induced of transcriptional activation of EMT-associated regulators [178]. (Fig. 3).

A growing number of studies indicated that the β2-adrenoceptor mediated signaling pathway contributes to tumor initiation and progression in human solid cancers, including in breast cancer [179], melanoma, prostate cancer [154], gastric cancer [180], and tongue squamous cell carcinoma (TSCC) [181]. The β2-adrenoceptor is a transmembrane, G protein-coupled receptor required for the response to adrenaline and noradrenaline. As shown on Fig. 3, activation of β2-adrenoceptor signaling can be functionally involved in EMT, contributing to invasion and metastasis in colon and gastric cancer and its deregulation in TSCC confirmed a role in EMT and TSCC tumor progression [181, 182].

More recently, our team provided new evidence suggesting the anti-tumor effects of the α-adrenoceptor blockade through modifications of the EMT phenotypic landscape within the tumor microenvironment. Molecular profiling identified the EMT effector, IGFBP-3 as the primary target for DZ-50, a novel quinazoline derivative of the α-adrenoceptor antagonist doxazosin [178]. Treatment of prostate and renal cancer cells with this new compound downregulates nuclear IGFBP-3, through TGF-β, consequently leading to the phenotypic re-differentiation of prostate cancer cells from EMT to MET and ultimately overcoming therapeutic resistance through lifting anoikis resistance [178, 183]. IGFBP-3 holds major clinical significance, as its expression is predictive of prostate cancer progression, and these studies provide the first evidence for IGFBP-3 as a target to treat therapeutically resistant prostate cancer [183]. (Fig. 3). Further studies aim to explore the role of the α-blockade and EMT reversal through IGFBP-3 in optimizing treatment strategies in both urologic tumors and benign disease.

Catecholamines can also contribute to the metastatic journey within the genitourinary tumor microenvironment of the primary tumor localization and or distant site. In prostate cancer, β-adrenoceptor signaling in osteoblasts promotes the secretion of CXCL12 and binding to CXCR4 on surface of prostate cancer cells resulting in increases bone metastasis formation [184]. Tumor-associated endothelial cells are also sensitive to catecholamines [4, 49, 172], consequently implicating targeting of tumor angiogenesis and metastatic spread by β-adrenoceptor antagonists. Recent evidence found that β-adrenoceptor signaling promotes growth of tumor-associated endothelial cells via increasing tumor cell access to oxygen and nutrients [4, 172]. Exposure of endothelial cells to the α1-adrenoceptor antagonist doxazosin led to significant inhibition of angiogenic properties (based on endothelial tube formation) [49]. Taken together these lines of evidence support the concept that the antiangiogenic activity of α- and β-adrenoceptor antagonists may prevent tumor progression and dissemination via reduced tumor perfusion and neovascularization.

While the pro-metastatic role of α- and β-adrenoceptors is still under intense investigation, it is not universal but rather disease- or cell-type specific [185–189]. As shown on Fig. 3, in the presence of agonist or hormone binding, β-adrenoceptor upregulates PKA activity, which in return, abolishes ERK signaling cascade and ultimately cancer cell proliferation and migration [185, 186]. A more recent study underscored the antitumor activity of α2A-aadrenoceptor in cervical cancer and suppression of cancer cell migration and invasion through inhibition of the PI3K/Akt/mTOR survival pathway [189]. The β-adrenoceptor antagonists are commonly used to treat hypertension and recent findings on the role of adrenergic signaling in tumor progression have stimulated drug repurposing of β-adrenoceptor antagonists as anticancer therapies. Several retrospective studies analyzed the outcome of breast cancer patients treated with β-adrenoceptor antagonists for hypertension [190–195]. Overall, these clinical studies highlight that use of β-adrenoceptor antagonists are associated with prolonged recurrence-free survival and overall survival in breast cancer patients. Recently, a phase II randomized trial is being conducted to evaluate the anti-metastatic properties of β-adrenoceptor antagonists (propranolol) in patients undergoing surgical resection of primary breast cancer [196]. Transcriptomic profiling of resected tumors treated with propranolol showed reduced expression of genes functionally involved in TGF-β-induced EMT including Snail, Slug and Smad (Fig. 3) [196, 197].

## Clinical landscape of targeting of adrenoceptor signaling in GU cancers

### Prostate cancer

Experimental studies in cell-based and preclinical models have reported that (with a quinazoline chemical structure) α-adrenoceptor antagonists induce apoptosis among the tumor epithelial cells, leading to significant suppression of prostate tumor growth, indicating that these medications may have therapeutic value in targeting prostate tumor dynamics for the treatment of prostate cancer patients with advanced disease [45].

The current clinical evidence on prostate cancer incidence in men treated with α-adrenoceptor antagonists for LUTS is conflicting. The lack of randomized trials in the area raises concerns in interpreting evidence, given the risk for detection bias caused by increased symptom-related care-contacts. Several large non-randomized studies have evaluated the incidence of prostate cancer in men treated with α-blockers due to LUTS. In a nationwide population-based case–control study in men diagnosed with prostate cancer from Sweden, evaluating 26 735 cases and 133 671 matched controls, results showed that men using α-adrenoceptor antagonists had an overall increased risk of prostate cancer (OR = 1.33, 95% CI = 1.27 to 1.39). [198]. However, the increased risk was only seen in men with Gleason Score 6–7, not in men with Gleason Score 8–10. The same pattern was observed in a nationwide cohort study of 3,009,258 men from Denmark [199]. Additionally, a Finnish prostate cancer screening trial showed no significant difference in the overall risk of prostate cancer incidence among those patients exposed to α-adrenoceptor antagonists was detected [200]. There was however a lower incidence of men with high-grade tumors in α-adrenoceptor users compared to non-users.

Epidemiological evidence derived from a larger retrospective cohort study of 249 986 men, of whom 7 764 were α-adrenoceptor antagonists, also showed a higher risk of diagnosis with Gleason Score 8–10 cancer in men exposed to α-blockade compared with those who were not; but an 11% lower risk of getting diagnosed with prostate cancer. However, the increased risk of high-grade prostate cancer in α-blocker users compared with non-users was not translated into a higher incidence of prostate cancer mortality [201]. Similarly, Sarkar et al. reported no effect on prostate cancer-specific mortality in men treated with α-adrenoceptor antagonists in their population-based cohort study of 80 875 men with stage I-IV prostate cancer within the Veterans Affairs health care system. Their data also contained information on α-blocker use, and their results show that 12-year cumulative incidence of prostate cancer -specific mortality and all-cause mortality was higher in 5α-reductase inhibitors (5-ARI) users, but no significant difference was seen in men treated with α-adrenoceptor antagonists. Further analyses showed that pre-diagnostic use of α-adrenoceptor antagonists was associated with a delayed diagnosis, which might explain the difference seen in mortality, again emphasizing the risk for detection bias [202].

We recognize that we must proceed with caution in the interpretation of the current evidence on prostate cancer incidence in men treated with α-adrenoceptor antagonists especially concerning causality due to the considerable risk for detection bias. It is also essential to recognize that prostate cancer rarely presents with obstructive voiding symptoms, decreasing the risk of missing the diagnosis due to the drug masking the symptoms [203]. Additionally, unlike 5-ARIs that suppress prostate specific antigen (PSA) by 50% after 3 months of use, there is no clinical evidence that α-blockers decrease PSA, which would reduce the risk of a delayed diagnosis. Moreover, Orsted et al. and Sarkar et al. performed studies to evaluate the effect of 5-ARI use and not α-adrenoceptor antagonists, which may introduce bias in the methodology [199, 202]. The current non-randomized evidence shows an overall non-significant effect of α-adrenoceptor antagonist use on both incidence and mortality, but the results in different risk-groups are conflicting. Ongoing retrospective studies and randomized trials are designed to evaluate the impact of α-adrenoceptor antagonists on prostate cancer incidence and mortality.

### Bladder cancer

On the basis of recent studies, manipulation of the adrenergic system for the purposes of therapeutic targeting has shown promise for the systemic treatment for bladder cancer. Current evidence has focused mainly on pharmacologic blockade of either α- or β-adrenergic receptors with in vivo and in vitro studies in pre-clinical models.

The quinazoline based α-adrenoreceptor antagonist group of drugs have been shown to have potential efficacy in both prevention and treatment of bladder cancer in patients. A retrospective observational study of 27,183 male patients in Kentucky linked to the SEER program of the NCI, was able to compare bladder cancer incidence in those exposed to quinazolone α1 blockade vs those not receiving this treatment [204]. Analysis of cumulative bladder cancer incidence was 0.24% in the α-blocker exposed vs 0.42% in the α-blocker unexposed group. This indicates that the number of men diagnosed with bladder cancer was 1.8 less per 1000 men in the exposed group, with a relative risk reduction of 43% for those exposed to alpha blockers [204]. Examination of bladder tumor tissue specimens post radical cystectomy using immunohistochemical methods to detect microvessel density and apoptosis, revealed that previous long-term exposure of patients to quinazolone α1 adrenoceptor, terazosin, had reduced tumor vascularity and increased apoptosis. Despite the obvious limitation of having only a small number of tumor specimens examined [9 cystectomy samples exposed vs 13 cystectomy samples unexposed to terazosin], this is consistent with the established apoptotic effect of terazosin on prostate cancer clinical specimens [205]. Similarly, in vitro studies assessing cell viability assays in human bladder cancer cell lines, found prazosin at concentrations of greater than 30microml/l was able to reduce cell viability and induce apoptosis [40]. In the same experiment, naftopidil, a piperazine based α1-adrenoceptor antagonist which is highly selective for α-1A and α-1D receptor subtypes (Fig. 2), was able to have the stronger effect in reducing bladder cancer cell viability at concentrations of 10-100microml/l [40]. Other studies have also showed orally administered naftopidil was able to reduce bladder tumor volume in mouse xenografts models via the activation of apoptosis [151]. In an in vitro study, doxazosin, a quinazoline based α-adrenoceptor antagonist, was able to inhibit the growth of HT137 bladder cancer cells, an effect that was reduced by pre-treatment with 5-hydroxytryptamine (5-HT, serotonin). This was thought to be due to the similar structure of adrenergic and 5-HT receptors [38, 206]. Interestingly, the expression of ELK1, a marker for tumor progression post radical cystectomy for bladder cancer, is inhibited by Silodosin, which also inhibits ELK1 bladder cancer cell survival and migration [207]. Based on this cell-derived evidence, Silodosin was proposed to potentiate the efficacy of cisplatin chemotherapy for patients with ELK1 bladder tumors, who have developed chemoresistance [38, 207].

Our current understanding of the neurobiology of cancer supports that β- adrenergic signaling influences tumorigenesis not only by landscaping the tissue phenotype within TME, but also impairing DNA repair mechanisms facilitating genomic instability [162]. Neural progenitor cells and sensory neurons can differentiate into adrenergic cells in the TME as a source of catecholamines [208, 209]. Further in vitro studies using murine cells pretreated with catecholamines identified a significantly increased DNA damage, an effect that was prevented by propranolol [210]. Growing evidence from rigorous molecular work has shown β-adrenergic signaling exerts a significant regulatory control on several cancer-promoting cellular processes including sustained proliferative signaling, resisting cell death, inducing cell migration, angiogenesis, immune evasion, inflammation, metabolic energy and TME landscape dynamics, all critical contributors to metastasis [162, 211]. Molecular profiling of bladder cancer pathobiology, has implicated the repurposing of propranolol as an anti-cancer agent with high therapeutic value in advanced stage tumors [212]. Moreover, there are trials due to start recruiting directly assessing propranolol as an adjuvant agent in combination with BCG for high risk non muscle invasive bladder cancer, assessing long term disease recurrence and progression and survival as outcomes (https://clinicaltrials.gov/ct2/show/NCT04493489).

### Renal cancer

The most common genetic abnormality for clear cell RCC (ccRCC) is the chromosome 3p deletion and inactivation of the von Hippel Lindau (VHL) tumor suppressor gene, present in almost all familial and up to 60% of sporadic RCCs [47, 213]. Loss of the VHL gene leads to the upregulation of hypoxia-inducible factor (HIF) and activation of vascular endothelial growth factor receptors (VEGFR), leading to tumorigenesis with an aggressive angiogenic phenotype [47, 213]. For patients with advanced localized renal cancer treatment with adjuvant VEGFR TKI sunitinib is approved by the FDA, although the modest clinical benefit and concern for the potential side effects has largely limited its clinical application to date [214]. For metastatic ccRCC, a number of approaches combining immune checkpoint inhibitors or with VEGFR-TKIs have now become the standard of care after demonstrating a definitive survival benefit in the first-line setting compared to sunitinib alone [215]. The current systemic therapeutic approaches include: targeting pathways of angiogenesis, immune checkpoint blockade, and mTOR inhibition. By inducing smooth muscle relaxation and vasodilation, these drugs are used for the treatment of hypertension (HTN) and obstructive symptoms associated with BPH (as discussed above) [216, 217]. This mechanism of action is also utilized in the treatment of renal and ureteric stones, as α1 blockers reduce intra-ureteral pressure and increase fluid passage [216].

As we discussed earlier adrenoceptor signaling has a well-established role in renal physiology. Immunohistochemical profiling shows the presence of both α- and β-adrenoceptors in the majority of kidney structures and cell types [215, 218]. Similar results are confirmed through in situ hybridization measurement of α- and β-adrenoceptor mRNA expression levels [219] (summarized on Fig. 1). These receptors play a role in many vital renal functions, rationalizing the presence of aberrant adrenoceptor signaling in a host of kidney diseases such as diabetic nephropathy and acute kidney injury [220, 221]. A molecular and immunohistochemical expression profile of β-adrenoceptors across a panel of different cancer types, revealed increased expression in clear cell RCC relative to normal kidney tissue [222]. At the translational setting, abnormal α- and β-adrenoceptor signaling is functionally linked to the pathophysiology of clinical hypertension, while epidemiological evidence has established a clear association between hypertension and RCC [223]. This provides a strong rationale supporting that modulation of adrenoceptor signaling using an α- or β-adrenoceptor antagonist may have therapeutic impact in RCC (Fig. 2).

Despite the widespread use of α-adrenoceptor antagonists among patients with RCC for comorbid conditions, direct analyses of the effect of these medications in RCC are limited to preclinical and observational studies. Cell-based studies support the anti-cancer effect of these drugs. Doxazosin and naftodipil, selective α1-adrenoceptor antagonists, inhibit the proliferation of RCC cells both in vitro and in vivo human tumor xenografts in mice [47, 213]. Propranolol induces loss of cell viability in RCC cells in vitro by increasing gene expression involved in apoptosis, decreasing HIF-α expression, and inhibiting the NFκB survival signaling [216, 217]. α-1 blockade has been linked to an increased RCC incidence, although the study is limited by confounding factors such as concurrent BPH or HTN [224]. On the other hand, the clinical evidence indicates that β-blockade is not associated with RCC risk in patients with HTN [225], and patients with resected localized RCC who use β-blockade did not have better outcomes [226, 227]. In a retrospective analysis of patients with metastatic RCC, concomitant use of β-blockers with immunotherapy is associated with improved clinical outcomes. However, this effect may be attributed to the blockade of β-adrenoceptor signaling on tumor immunosuppression [228, 229].

Given the recent evidence implicating both α- and β-adrenoceptors as functional contributors to RCC pathogenesis and progression, further translational studies are warranted to better understand the targeting value of the adrenergic signaling in advanced RCC and overcoming resistance to current therapeutic modalities.

## Impact of pharmacologic α- and β-adrenergic blockade on quality of life

Studies that examined the impact of the pharmacologic α- and β-adrenoceptor antagonists on patient quality of life and functional status focused primarily on patient populations with cardiovascular diseases (e.g., HTN, cardiovascular diseases) and BPH. Although shown to be beneficial in primary and secondary prevention of cardiovascular diseases, and the reduction of reduced symptom bother in BPH patients, concerns about the side effects of β-adrenoceptor antagonists were reported by patients and physicians because of their impact on patient sexual function [230]. Further clinical evidence suggests that β-adrenoceptor antagonists disrupt the balance between α- and β-adrenergic nerve fibers thus leading to increased deterioration of sexual function/ erectile dysfunction in men [231]; the molecular mechanisms underlying this effect remain unclear. Similarly, among the side effects commonly reported with the use of β-adrenoceptor antagonists in these patient populations, are symptoms related to the central nervous system including fatigue, reduced cognitive function, and lethargy leading to poor treatment compliance [232]. Evidence from clinical studies also demonstrated the effects of β-adrenoceptor antagonists on mood state and depression [233–236], as well as with increased sleep disturbances [237, 238], sleep quality [rapid eye movement (REM) and non-REM sleep], and increased risk of sleep apnea, thus further contributing to fatigue, reduced cognitive function and quality of life [239].

As discussed above, the increasing use of pharmacologic α- and β-adrenoceptor inhibition is well documented in patients with urologic disease including kidney stones, BPH, and bladder, prostate, and renal cancer with side effects varying by the disease population [38, 40, 43, 46, 47]. The impact of pharmacologic α- and β-adrenoceptor antagonists on patient quality of life and functioning status among kidney stones, BPH, and bladder, prostate, and renal cell cancer patients, has revealed a significant effect on increasing heart palpitation, fatigue, dizziness, and increased risk for erectile dysfunction and renal cell carcinoma independent of hypertension [224].

## Conclusions

In summary, this review discusses the promise of repurposing of the α- and β-adrenoceptor antagonists for the treatment of benign and malignant urologic diseases. α1-adrenoceptor antagonists may be of value in the treatment of GU malignancies as anti-tumor-modalities as well as in the therapeutic management of benign urologic conditions such as obstructing kidney stones and voiding function. Retrospective epidemiological studies are underway to assess the impact of α1 adrenoceptor antagonists as chemopreventive agents, and prospective clinical trials designed to investigate their efficacy in pre-surgical, post-surgical, and in-patient settings of metastatic disease. Ongoing research efforts exploit the action of α- and β-adrenoceptor signaling mechanisms at: (a) the cellular level, by interrogating the functional exchanges between apoptosis/anoikis signaling and phenotypic configurations within the prostate, bladder and kidney tumor microenvironment; and (b) the clinical setting by undertaking retrospective epidemiological studies and prospective clinical trials to determine the therapeutic value of α1- and β-adrenoceptor antagonists in patients with metastatic disease.

The present study not only describes the evidence on the beneficial effects of α- and β- adrenoceptor antagonists in long-term sequelae of AKI and progression of CKD, but also recognizes the adverse outcomes in kidney function like GFR and RBF, despite BP- and/or blood glucose-lowering effects. Furthermore the α- and β adrenoreceptor antagonists have distinct effects depending on tissue or injury, that would be exploited for tissue-targeted approaches using conditional genetic ablation and cell targeted drug delivery systems. The analytical insights presented here are of high relevance in understanding the molecular and phenotypic effects of pharmacologic action of clinically available adrenoceptor antagonists and their targeting of the α- and β- adrenoceptor subtypes in kidney, bladder and prostatic disease.

## Data Availability

Data sharing is not applicable to this article as no datasets were generated or analyzed during the current study.

## Abbreviations

5-ARI: 5α-Reductase inhibitor
5-HT: 5-Hydroxytryptamine
ADRA1D: Adrenoceptor α1D
AKI: Acute kidney injury
Akt: Protein kinase B
Ang: Angiotensin
Ang II: Angiotensin II
BAD: Bcl-2-associated death promotor
BCG: Bacillus Calmette–Guerin
Bcl-2: B-cell lymphoma 2
BPH: Benign prostatic hyperplasia
cAMP: Cyclic adenosine monophosphate
ccRCC: Clear cell renal cell carcinoma
CDK: Cyclin-dependent kinase
CDK1: Cyclin-dependent kinase 1
CI: Confidence interval
CKD: Chronic kidney disease
Coa6: Cytochrome c oxidase assembly factor 6
CREB: CAMP responsive element binding protein
CRPC: Castration-resistant prostate cancer
DAG: Diacylglycerol
DNA: Deoxyribonucleic acid
dUTP: Deoxyuridine triphosphate
ECM: Extracellular matrix
EMT: Epithelial-mesenchymal transition
ESKD: End stage kidney disease
FADD: Fas-associated death domain
FAK: Focal adhesion kinase
FDA: Food and Drug Administration
FGF: Fibroblast growth factor
GFR: Glomerular filtration rate
GU: Genitourinary
HIF: Hypoxia-inducible factor
HIF1: Hypoxia-inducible factor 1
HIF-1α: Hypoxia-inducible factor α
HPA: Human Protein Atlas
HTN: Hypertension
IGFBP-3: Insulin-like growth factor-binding protein-3
IL-6: Interleukin 6
ILK: Integrin-linked kinase
IRI: Ischemia/reperfusion injury
LUTS: Lower urinary tract symptoms
MAPK: Mitogen-activated protein kinase
MET: Mesenchymal-epithelial transition
mRNA: Messenger RNA
mTOR: Mammalian target of rapamycin
NCI: National Cancer Institute
NE: Norepinephrine
NO: Nitric oxide
NOS: Nitric oxide synthase
OAB: Overactive bladder
OR: Overall risk
PCa: Prostate cancer
PI3-kinase: Phosphatidylinositol 3-kinase
PKA: Protein kinase A
PSA: Prostate specific antigen
RAS: Renin-angiotensin system
RBF: Renal blood flow
RCC: Renal cell carcinoma
REM: Rapid eye movement
RNA: Ribonucleic acid
STAT3: Signal transducer and activator of transcription 3
TGF-α: Transforming growth factor α
TGF-β: Transforming growth factor-β
TGF-β1: Transforming growth factor-β1
TSCC: Tongue squamous cell carcinoma
TKI: Tyrosine kinase inhibitor
TME: Tumor microenvironment
TNF-α: Tumor necrosis factor α
UUO: Ureteral obstruction
TUNEL: Terminal deoxynucleotidyl transferase-mediated dUTP nick-end labeling
VEGF: Vascular endothelial growth factor
VEGF: Vascular endothelial growth factor receptor
VHL: Von Hippel Lindau
